# Mucocutaneous Manifestations Reported by Inflammatory Bowel Disease Patients in University Hospital

**DOI:** 10.15388/Amed.2024.31.1.23

**Published:** 2024-02-27

**Authors:** Ieva Renata Jonaitytė, Vita Karpavičiūtė, Gediminas Kiudelis, Juozas Kupčinskas, Laimas Jonaitis

**Affiliations:** 1 Department of Gastroenterology, Lithuanian University of Health Sciences, Kaunas, Lithuania; 2 Center of Dermatovenerology, Vilnius University Hospital Santaros klinikos, Vilnius, Lithuania

**Keywords:** ulcerative colitis, Crohn’s disease, extraintestinal manifestation, skin lesions, mucocutaneous lesions, opinis kolitas, Krono liga, ekstražarniniai pažeidimai, odos ir gleivinės pažeidimai

## Abstract

**Background:**

Inflammatory bowel disease (IBD) may affect organs outside the intestines, it is called extraintestinal manifestations of IBD. Data on the prevalence of mucocutaneous manifestations in IBD patients are very limited, therefore, the aim of this study was to assess the prevalence of skin and mucosal lesions and to determine the relationship with demographic factors, clinical features, and systemic treatment.

**Materials and methods:**

Prospective study included 162 out-patients with IBD who were managed in the tertiary care center. Ulcerative colitis (UC) was diagnosed in 117 patients, Crohn‘s disease (CD) in 45. Patients completed the questionnaire containing demographic and IBD data, questions about mucocutaneous lesions (in past or present state).

**Results:**

Overall mucocutaneous lesions were reported by 48.1% of IBD patients. Skin lesions were reported by 40.7% of patients, oral mucosal lesions were reported by 16.7%, without significant differences between sexes or IBD types. In 47 (29%) of patients, skin lesions appeared together with IBD or during the course of the disease. The most common skin lesions were psoriasis (8.0%), erythema nodosum (5.6%), pyoderma gangrenosum and acne (3.7% each). UC patients mostly reported about psoriasis (9.4%), while CD patients about erythema nodosum (11.1%). There were more frequent skin lesions in patients with more extensive UC type (p = 0.01), while no difference was noticed between different types of CD. The average duration of IBD in patients with skin lesions was similar to those without lesions (9.3±6.7 vs. 9.4±6.7 years).

**Conclusions:**

Mucocutaneous lesions were reported by 48.1% of inflammatory bowel disease patients. The frequency of mucocutaneous lesions does not differ significantly between UC and CD, and a longer duration of illness is not a predictive factor for the appearance of lesions. More extensive UC is related to higher frequency of skin lesions.

## Abstract (it)

**Įvadas:**

Uždegiminių žarnų ligų (UŽL) metu gali atsirasti vadinamųjų ekstražarninių pažeidimų, kurie apima odą, sąnarius, akis ir kitus organus. Duomenų apie odos ir gleivinių pažeidimų paplitimą sergant UŽL labai trūksta, todėl šio tyrimo tikslas buvo įvertinti odos ir gleivinių pažeidimų paplitimą bei nustatyti jų ryšį su demografiniais veiksniais, klinikiniais požymiais ir sisteminiu gydymu.

**Medžiaga ir metodai:**

Prospektyviajame tyrime dalyvavo 162 UŽL sergantys pacientai, kurie kreipėsi gydytojo gastroenterologo konsultacijos į tretinio lygio sveikatos priežiūros centrą. Opinis kolitas (OK) diagnozuotas 117 pacientų, Krono liga (KL) – 45 pacientams. Anketinės apklausos bždu surinkti duomenys apie tiriamųjų demografinius ir UŽL klinikinius duomenis, odos ir gleivinių pažeidimus (buvusius ir esamus).

**Rezultatai:**

Iš viso odos ir gleivinių pažeidimai nustatyti 48,1 proc. UŽL ligonių. Apie odos pažeidimus pranešė 40,7 proc. pacientų, esant burnos gleivinės pažeidimų nurodė 16,7 proc. Reikšmingų skirtumų lyginant pagal lytį ar UŽL tipą nenustatyta. 47 (29 proc.) pacientams odos pažeidimų atsirado kartu su UŽL arba ligos eigoje. Dažniausiai pacientų nurodyti odos pažeidimai buvo psoriazė (8,0 proc.), mazginė eritema (5,6 proc.), gangreninė pioderma ir aknė (po 3,7 proc.). OK sergantys pacientai dažniausiai pranešė apie žvynelinę (9,4 proc.), o sergantieji KL – apie mazginę eritemą (11,1 proc.). Pacientams, kuriems OK buvo labiau išplitęs žarnyne, odos pažeidimų buvo dažniau (p = 0,01), o skirtumų tarp skirtingų KL tipų nenustatyta. Vidutinė UŽL trukmė pacientų, turinčių odos pažeidimų, buvo panaši kaip ir jų neturinčiųjų (9,3±6,7 ir 9,4±6,7 metų atitinkamai).

**Išvados:**

48,1 proc. pacientų, sergančių uždegimine žarnyno liga, pranešė apie gleivinės ir odos pažeidimus. Odos ir gleivinės pažeidimų dažnis sergančių OK ir KL pacientų reikšmingai nesiskiria, o ilgesnė ligos trukmė nėra prognostinis veiksnys, nurodantis didesnę pažeidimų atsiradimo riziką. Labiau žarnyne išplitęs OK yra susijęs su didesniu odos pažeidimų dažniu.

## Introduction

In the 21st century, inflammatory bowel diseases (IBD) – ulcerative colitis and Crohn‘s disease – have become global diseases, and although the incidence in Western countries tends to stabilize, the incidence in newly developed industrialized countries, whose society is becoming more and more westernized, including Lithuania, is still increasing [1-4]. IBD can affect not only the digestive tract, but also other organs outside the intestines. These lesions are called extraintestinal manifestations (EIMs) of IBD. In both Crohn‘s disease (CD) and ulcerative colitis (UC), EIMs most commonly involve the musculoskeletal system, skin, hepatobiliary tract, and eyes. The frequency of different EIMs in IBD patients ranges from <1% up to 50% [5-6].

According to different authors, skin and mucosal lesions occur in 6–53% of patients with IBD [6-9]. The most common lesions associated with IBD are erythema nodosum, pyoderma gangrenosum, and oral mucosal lesions [10]. Based on pathogenesis and relationship to the underlying intestinal disease, mucocutaneous lesions can be divided into five groups: specific manifestations, disorders associated with IBD, reactive manifestations, conditions secondary to the treatment and conditions caused by nutritional malabsorption [11].

There is increasing evidence of secondary mucocutaneous diseases induced by IBD treatment with TNF-α antagonists [12, 13]. These lesions include local and systemic adverse reactions, paradoxical skin lesions (typically anti-TNF agents are approved for the treatment of these disorders, for example, psoriasis), and infectious complications [11, 13]. The increased incidence of skin cancer with anti-TNF use is still controversial [14, 15]. In a meta-analysis performed by Nigam et al. [13], the overall incidence of any dermatological reaction in patients with IBD treated with anti-TNF therapy was 19.4%, while the most common event (in 39 studies) was psoriasis/psoriatic rash with an incidence of 5.6%. A retrospective study conducted by Weizman et al. [16] found that the median time to onset of psoriasis was 19 months after initiation of anti-TNF, with an earlier onset of psoriasis in patients treated with adalimumab versus infliximab (15 vs. 26 months).

We would like to emphasize, that the data on the prevalence of mucocutaneus manifestation in IBD patients are limited, different numbers on the prevalence of these lesions are usually derived from small series and may be possibly biased. We could find only a few well-designed clinical trials [6-9, 17, 18]. Therefore, the aim of our study was to assess the prevalence of skin and mucosal lesions in IBD patients at University hospital and to determine the relationship of lesions with demographic factors, clinical features, and systemic treatment.

## Materials and methods

### Study design and participants

The prospective study was conducted in 2022 from January to October. Out-patients with IBD who were managed in the hospital of Lithuanian University of Health Sciences Kaunas Clinics were included in this study. A questionnaire survey of the subjects was directly applied during the consultation of the gastroenterologist. A total of 162 IBD patients agreed to answer to the survey questions.

The inclusion criteria were age ≥18 years, history of UC or CD, voluntary participation, ability to answer the questionnaire independently.

According to the time of appearance, skin lesions were divided into two groups: 1) those appeared before the diagnosis of IBD; 2) appearing together with IBD or during the course of the disease. Our results and analysis were made using all cases of skin and mucosal lesions. The relationship of skin lesions with systemic drugs for the treatment of IBD was analyzed by evaluating skin lesions that appeared during the course of IBD. We must note that the assessment of the relationship of used medications with skin or mucous lesions and some other parameters was based on the opinion of the patients.

### Ethical consideration

This study was conducted in accordance with the Declaration of Helsinki and approved by the Lithuanian University of Health Sciences Bioethics Center, study No. BEC-MF-168, notification of determination received on 12 Jan 2022. Additionally, informed consent was obtained from all subjects involved in this study. The confidentiality and anonymity of the participants’ responses were guaranteed.

### Statistical analysis

Data were tabulated in Microsoft Excel, and statistical analyses were performed using IBM SPSS Statistics for Windows v25.0. Continuous variables were expressed as mean ± standard deviation for those conforming to a normal distribution. Categorical variables were expressed as frequency (%). The Chi-square test and Fisher’s exact test were used to compare the frequency of skin and mucosal lesions between UC and CD patient groups (subgroups) and between men and women. The Mann–Whitney U test was used to compare the duration of IBD between subjects with and without skin lesions. A p-value < 0.05 was deemed statistically significant.

## Results

### Characteristics of patients

A total of 162 patients were included in the study, with a mean age (MA) of all patients of 42.5 ±14.2 years, without significant difference between sexes. The youngest patient was 19 years old, and the oldest was 75 years old. Ulcerative colitis was diagnosed in 117 (72.2%) patients: 17 (14.5%) had a proctitis, 30 (25.6%) had left-sided colitis, and 70 (59.8%) had a pancolitis. A total of 45 (27.8%) patients had Crohn’s disease – including 17 (37.8%) with ileitis, 11 (24.4%) patients with colitis, and 17 (37.8%) with ileocolitis. The majority of patients (95.7%) were using systemic treatment for IBD, such as oral mesalazine and/or azathioprine alone or in combination with glucocorticoids and/or biological therapy. Additionally, some patients were prescribed JAK inhibitors (either as monotherapy or in combination with mesalazine/prednisolone). Additional demographic and IBD information are presented in Table 1.

**Table 1:** Demographic and general IBD data.

	Total	UC	CD
**Patient count**	162 (100 %)	117 (72.2%)	45 (27.8%)
*Male*	93 (57.4%)	67 (57.3%)	26 (57.8%)
*Female*	69 (42.6%)	50 (42.7%)	19 (42.2%)
**Mean age (SD)**	42.5 (14.2)	43.1 (14)	40.9 (14.9)
**Illness duration in years (SD)**	9 (6.8)	9.5 (7.0)	7.7 (6.1)
*<5 years*	46 (28.4%)	31 (26.5%)	15 (33.3%)
*5–10 years*	53 (32.7%)	35 (29.9%)	18 (40%)
*>10 years*	63 (38.9%)	51 (43.6%)	12 (26.7%)
**Used systemic treatment**	155 (95.7%)	112 (95.7%)	43 (95.6%)
**Biological therapy**	82 (50.6%)	50 (42.7%)	32 (71.1%)
*Infliximab/adalimumab*	46 (56.1%)	25 (50.0%)	21 (65.6%)
*Vedolizumab*	13 (15.9%)	11 (22.0%)	2 (6.3%)
*Ustekinumab*	9 (11.0%)	3 (6.0%)	6 (18.8%)
*Mirikizumab (clinical trial)*	11 (13.4%)	8 (16.0%)	3 (9.4%)
*Risankizumab (clinical trial)*	3 (3.7%)	3 (6.0%)	0 (0.0%)
**JAK inhibitors**	6 (3.7%)	5 (4.27%)	1 (2.22%)
*Tofacitinib*	4 (66.7%)	4 (80.0%)	0 (0%)
*Upadacitinib (clinical trial)*	2 (33.3%)	1 (20.0%)	1 (100%)

* IBD – inflammatory bowel disease; UC – ulcerative colitis; CD – Crohn’s disease; SD – standard deviation; JAK inhibitors – Janus kinase inhibitor.

### Skin and mucosal lesions

Skin lesions were reported by 66 (40.7%) patients, with some indicating more than 1 skin lesion. Among them, 37 (39.8%) were men and 29 (42.0%) were women, p > 0.05. Skin lesions occurred in 51 (43.6%) UC group and in 15 (33.3%) CD group patients, p > 0.05. The subjects indicated the following skin lesions: psoriasis (8.0%), erythema nodosum (5.6%), pyoderma gangrenosum and acne (3.7% each), atopic dermatitis (2.5%), vitiligo and seborrheic dermatitis (1.9% each), epidermolysis bullosa acquisita and vasculitis (0.6% each), unspecified dermatitis (6.8%) and others (10.5%). The most common skin lesions among patients with UC were psoriasis (9.4%), pyoderma gangrenosum and acne (4.3% each), while the most common lesions among CD patients were erythema nodosum (11.1%) and psoriasis (4.4%). The detailed distribution of skin lesions among IBD patients is presented in Table 2.

**Table 2:** The prevalence of skin lesions reported by inflammatory bowel disease patients.

Skin lesions	Total n = 162	Ulcerative colitis n = 117	Crohn’s disease n = 45	p value
Psoriasis	13 (8.0%)	11 (9.4%)	2 (4.4%)	0.473
Erythema nodosum	9 (5.6%)	4 (3.4%)	5 (11.1%)	0.126
Pyoderma gangrenosum	6 (3.7%)	5 (4.3%)	1 (2.2%)	0.877
Acne	6 (3.7%)	5 (4.3%)	1 (2.2%)	0.877
Vitiligo	3 (1.9%)	3 (2.6%)	0 (0%)	0.665
Epidermolysis bullosa acquisita	1 (0.6%)	1 (0.9%)	0 (0%)	1.000
Vasculitis	1 (0.6%)	1 (0.9%)	0 (0%)	1.000
***Others:***				
Seborrheic dermatitis	3 (1.9%)	3 (2.6 %)	0 (0%)	0.665
Atopic dermatitis	4 (2.5%)	4 (3.4%)	0 (0%)	0.126
Unspecified dermatitis	11 (6.8%)	8 (6.8%)	3 (6.7%)	1.000
Others skin lesions	17 (10.5%)	13 (11.1%)	4 (8.9%)	0.899

Reported oral mucosal lesions were aphthous stomatitis or isolated aphthae. It has been indicated by 27 (16.7%) subjects – the prevalence was slightly higher in women than men, respectively, 15 (21.7%) versus 12 (12.9%), p>0.05. These disorders occurred in 17 (14.5%) UC and 10 (22.2%) CD patients, p>0.05.

After evaluating the total number of patients with skin and/or mucosal lesions, it was determined that 78 (48.1%) of the patients participating in this study reported mucocutaneous lesions: isolated skin lesions occurred in 51 (31.5%) patients, 15 (9.3%) patients had both skin and mucosal lesions, while 12 (7.4%) respondents reported isolated oral mucosal lesions.

### The prevalence of mucocutaneous lesions in different age groups

In order to evaluate possible differences in the prevalence of mucocutaneous lesions depending on age, participants were divided into two groups: those aged up to 40 years (n = 77) and those older (n = 85). The detailed data are presented in Table 3. The findings revealed that overall, skin lesions are more prevalent among younger individuals (p = 0.003). However, when examining specific skin conditions separately, it was observed that the prevalence of separate skin lesions is not statistically different. Analyzing the data of UC and CD groups separately, we found that in UC group skin lesions were more prevalent in younger patients (respectively, 32 (59.3%) versus 19 (30.2%), p = 0.003), while in CD patients there were no significant differences (respectively, 9 (39.1%) versus 6 (27.3%), p > 0.05). Meanwhile, oral mucosal lesions manifested equally in both age groups, regardless of the type of IBD.

**Table 3:** The prevalence of mucocutaneous lesions reported by patients with IBD by age groups.

Mucocutaneous lesions	Total n = 162	≤ 40 years old n = 77	≥ 41 years old n = 85	p value
Patients with skin lesions	66 (40.7%)	41 (53.2%)	25 (29.4%)	p = 0.003
Oral mucosal lesions	27 (16.7%)	14 (18.2%)	13 (15.3%)	p = 0.778
Psoriasis	13 (8.0%)	8 (10.4%)	5 (5.9%)	p = 0.444
Erythema nodosum	9 (5.6%)	5 (6.5%)	4 (4.7%)	p = 0.737
Pyoderma gangrenosum	6 (3.7%)	2 (2.6%)	4 (4.7%)	p = 0.684
Acne	6 (3.7%)	5 (6.5%)	1 (1.2%)	p = 0.103
Vitiligo	3 (1.9%)	0 (0%)	3 (3.5%)	p = 0.247
Epidermolysis bullosa acquisita	1 (0.6%)	1 (1.3%)	0 (0%)	p = 0.475
Vasculitis	1 (0.6%)	1 (1.3%)	0 (0%)	p = 0.475
***Others:***				
Seborrheic dermatitis	3 (1.9%)	2 (2.6%)	1 (1.2%)	p = 0.605
Atopic dermatitis	4 (2.5%)	4 (5.2%)	0 (0%)	p = 0.050
Unspecified dermatitis	11 (6.8%)	7 (9.1%)	4 (4.7%)	p = 0.426
Others skin lesions	17 (10.5%)	11 (14.3%)	6 (7.1%)	p = 0.214

* IBD – inflammatory bowel disease. *Note* – some patients reported more than 1 skin lesion.

### The relationship between skin and mucosal lesions with extension of IBD

**Figure 1:**
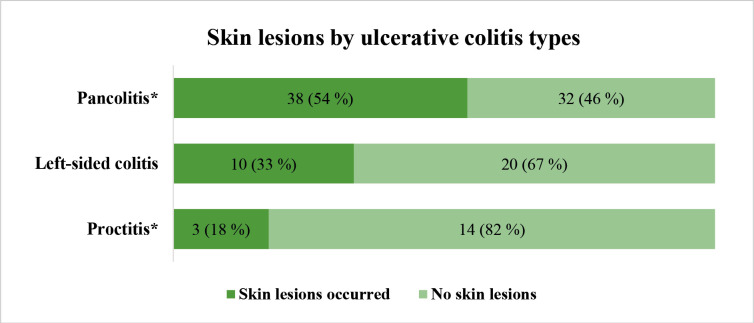
Skin lesions distribution between ulcerative colitis types. * p = 0.01 between the proctitis and pancolitis groups.

Depending on the extension of UC, it was found that skin lesions more frequently occur in pancolitis patients compared to proctitis group (p = 0.01). The detailed distribution of skin lesions between UC groups is presented in Figure 1.

Lesions of the oral mucosa were reported by 3 of 17 (17.6%) proctitis patients, 6 of 30 (20.0%) patients with left-sided colitis and 8 of 70 (11.4%) patients suffering from pancolitis, p>0.05

When comparing different types of CD, skin lesions were reported by 3 of 17 (17.6%) patients with ileitis, 5 of 11 (45.5%) patients with colitis and 7 of 17 (41.2%) patients with ileocolitis. The oral mucosal lesions occurred in 4 of 17 (23.5%) patients with ileitis, 3 of 11 (27.3%) colitis and 3 of 17 (17.6%) patients with ileocolitis. In contrast to UC, the incidence of skin and mucosal lesions was not statistically significantly different between different types of CD.

### The relationship between skin and mucosal lesions with duration of IBD

The average duration of IBD in subjects who reported skin lesions was similar to those without lesions (9.3 ±6.7 vs. 9.4 ±6.7 years, respectively), p>0.05. The same trend is observed with lesions of the oral mucosa: the average duration of IBD in lesions group was 10.2 ±8.9 years, comparing with those without lesions 9.2 ±6.1 years, p>0.05. Also, there were no significant differences when comparing groups of UC and CD patients separately. The frequency of mucocutaneous lesions does not depend on the duration of the IBD, and a longer duration of illness is not a predictive factor for the appearance of mucocutaneous lesions.

### The relationship between skin lesions with the time of IBD diagnosis

**Figure 2:**
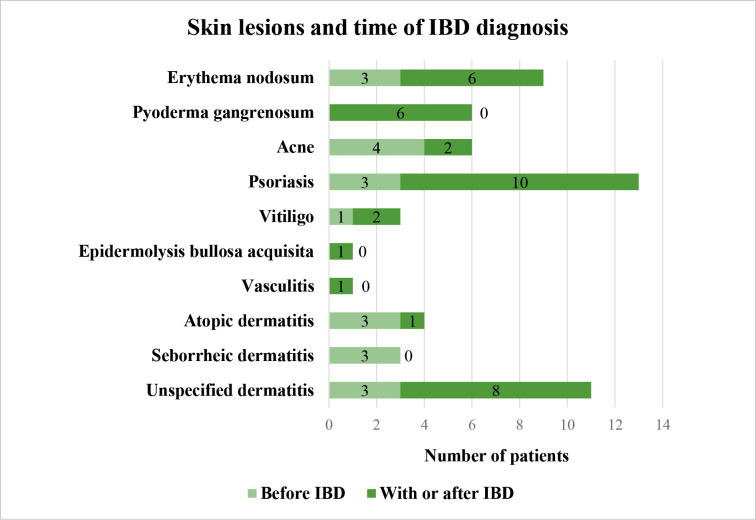
The distribution of skin lesions according to the time of their onset, p > 0.05 * IBD – inflammatory bowel disease.

According to the time of appearance, skin lesions can be divided into two groups – those that appeared before the diagnosis of IBD and those that were diagnosed together with IBD or during its course. Analyzing 66 patients who reported about skin lesions, 47 (71.2%) of them indicated that skin lesions appeared together with the onset of IBD or during the course of the disease, while 19 (28.8%) of subjects had skin lesions before the diagnosis of IBD. The detailed distribution of skin lesions according to the time of onset is presented in Figure 2.

### The relationship of skin lesions and drugs used for the treatment of IBD

Skin lesions in total were reported by 37 of 82 (45.1%) patients treated with biological therapy compared with 29 of 80 (36.3%) in the nonbiological treatment group, with no statistically significant difference between these two groups, p > 0.05 (however, data on the occurrence of skin lesions before or after the onset of biological treatment were not collected in this study). The most common skin lesions among patients using biological therapy were unspecified dermatitis, pyoderma gangrenosum, psoriasis and erythema nodosum.

According to the patients’ opinion, 16 of 66 (24.2%) subjects observed an association between exacerbation of skin lesions and drugs used to treat IBD. The exacerbation of skin lesions, possibly caused by biological therapy, was indicated by 10 of 66 (15.2%) patients, caused by azathioprine – 6 of 66 (9.1%), prednisolone – 4 of 66 (6.1%) patients. Comparing UC and CD patients, worsening of skin symptoms was slightly more often reported by patients in the CD group, although there was no statistically significant difference (33.3% vs. 21.6%), p > 0.05.

## Discussion

We found that the frequency of skin lesions among IBD patients in our series is 40.7%, and the overall frequency of skin–mucosal lesions is 48.1%. According to the data of previous studies, the frequency of skin and mucosal lesions varies from 5.8% to 53.3% [7-9]. The differences between the results can be explained by the fact that different skin lesions were evaluated. In this study, we analyzed all skin lesions reported by patients with IBD. It is also important to note that we analyzed patients treated at the tertiary care center. Possibly, these patients are more resistant to treatment, with more severe forms of the disease and hypothetically more frequent extraintestinal, including mucocutaneous, manifestations. We would like to stress, that there are very limited data and we hardly could find well designed prospective epidemiological studies regarding the skin lesions in IBD patients.

All cases of skin and mucosal lesions were analyzed in this study, but it is theoretically possible to assume that the real skin lesions associated with IBD are those lesions that were diagnosed together with IBD onset or during the course of IBD: in this case skin lesions frequency is 29%. However, considering that some skin lesions may occur before the diagnosis of UC or CD, we preferred a detailed analysis of all skin lesions.

In our study, the most common skin lesion among IBD patients was psoriasis (8%). This condition was slightly more common among UC (9.4%) than CD (4.4%), but not statistically significant. In a systematic review and meta-analysis conducted by Alinaghi et al. [19] the frequency of psoriasis among IBD patients was found to be half as low (4.2%) and, in contrast to our study, psoriasis was slightly more frequent among CD patients (3.6%) than UC (2.8%) patients. Two studies conducted in Turkey and Greece found psoriasis to be more common in UC than in CD patients, but a statistically significant difference was also not reached [7, 8]. Psoriasis or psoriatic rash may occur as a paradoxical reaction in patients treated with TNF-α inhibitors [16, 20-22]. Therefore, a slightly higher frequency of psoriasis in our study could possibly be related to the fact that a large proportion of patients (50.6%) were treated with biological therapy, while 56.1% of these patients were treated specifically with TNF-α inhibitors. (Although in this study data about previous treatment with biological drugs was not collected, in our center, TNF-α inhibitors remain one of the first-line biological agents for IBD treatment. Therefore, it cannot be ruled out that the rest of this group patients probably were previously treated with TNF -α inhibitors). Psoriasis caused by these medications occurs in 1.1–7.3% of IBD patients [22].

We found that the frequency of erythema nodosum among IBD patients is 5.6% and this condition is more common in CD (11.1%) than in UC (3.4%), p>0.05. In the Greek study [7], the incidence of erythema nodosum was almost the same as in our study (5.3%), and similarly more frequently affected CD group patients. In the study performed by Ampuero et al. [18], the incidence of erythema nodosum (7.1%) was slightly higher than our results.

In our study, the incidence of pyoderma gangrenosum among IBD patients was 3.7%. This skin condition was reported more often by UC than CD patients (4.3% vs. 2.2%, p > 0.05). According to other authors, the frequency of pyoderma gangrenosum is slightly lower and reaches 0.8–2.3% [6, 7]. In a Turkish study, similarly to our study, pyoderma gangrenosum was more common among patients with UC [8].

According to our data, lesions of the oral mucosa affected 16.7% of patients. These lesions were more frequent among CD (22.2%) than UC (14.5%) patients, p > 0.05. We did not collect data about separate oral mucosal lesions. In a Turkish study, aphthous stomatitis (40.2%) was the most common of all mucocutaneous lesions and occur more frequently in UC (44.6%) than CD (33.3%) patients [8]. While in the study conducted in Greece, the incidence of aphthous stomatitis was only 6.1% and it affected more often CD (6.9%) than UC (5.2%) patients [7].

In this study, we found that the frequency of mucocutaneous lesions does not depend on the duration of the IBD, and a longer duration of illness does not lead to a higher frequency of lesions. In UC patients, skin lesions are more common in the more extended form of the disease, it was found that skin lesions statistically significantly more frequently occur in pancolitis patients compared to proctitis group. This might be explained that the larger extent of mucosal inflammation is related with more extensive inflammatory response in different parties of the human body and the skin inclusively. In CD the frequency of mucocutaneous lesions does not differ significantly between different forms, though it has some trends to be more frequent in CD involving colon. A Turkish study also found no association between the frequency of skin and mucosal lesions and duration of IBD or the different forms of CD. However, in contrast to our study, no associations were found between the frequency of skin lesions and the extension of UC [8]. The study by Ampuero and co-authors [18] also found no association with extension of IBD.

We have to recognize that our study has some disadvantages as it was based on patients completed data and the sample size is not very large. Nevertheless, the prospective design of our research must be considered as an advantage and data can be considered as confident. We think that our data could add the significant information on this topic as there are too little high-quality data. It is clear, that there is an urgent need to investigate this issue in large scale prospective well-designed studies.

## Conclusions

In conclusion, it was found that overall mucocutaneous lesions were reported in 48.1% of inflammatory bowel disease patients in Lithuanian tertiary care University center. The frequency of mucocutaneous lesions does not differ significantly between ulcerative colitis and Crohn’s disease, and a longer duration of illness is not a predictive factor for the appearance of mucocutaneous lesions. The larger extension of ulcerative colitis leads to a higher frequency of skin lesions, while in Crohn’s disease the frequency of skin lesions did not depend on the type of the disease.

## References

[1] GBD 2017 Inflammatory Bowel Disease Collaborators (2020). The global, regional, and national burden of inflammatory bowel disease in 195 countries and territories 1990-2017: a systematic analysis for the Global Burden of Disease Study 2017. Lancet Gastroenterol Hepatol.

[2] Kiudelis G, Jonaitis L, Adamonis K, Žvirblienė A, Tamelis A, Kregždytė R, Kučinskienė R, Šventoraitytė J, Kupčinskas L (2012). Incidence of inflammatory bowel disease in Kaunas region, Lithuania. Medicina (Kaunas).

[3] Ng SC, Shi HY, Hamidi N (2017). Worldwide incidence and prevalence of inflammatory bowel disease in the 21st century: a systematic review of population-based studies. Lancet.

[4] Karpavičiūtė V, Kiudelis G, Kupčinskas J, Kupčinskas L (2023). Trends in the Prevalence of Inflammatory Bowel Disease in Lithuania during 2001-2020. J Crohn‘s Colitis.

[5] Rogler G, Singh A, Kavanaugh A, Rubin DT (2021). Extraintestinal Manifestations of Inflammatory Bowel Disease: Current Concepts, Treatment, and Implications for Disease Management. Gastroenterology.

[6] Vavricka SR, Rogler G, Gantenbein C (2015). Chronological Order of Appearance of Extraintestinal Manifestations Relative to the Time of IBD Diagnosis in the Swiss Inflammatory Bowel Disease Cohort. Inflamm Bowel Dis.

[7] Karmiris K, Avgerinos A, Tavernaraki A (2016). Prevalence and Characteristics of Extra-intestinal Manifestations in a Large Cohort of Greek Patients with Inflammatory Bowel Disease. J Crohns Colitis.

[8] Topaloğlu Demir F, Kocatürk E, Yorulmaz E, Adalı G, Kavala M (2014). Mucocutaneous manifestations of inflammatory bowel disease in Turkey. J Cutan Med Surg.

[9] Zippi M, Corrado C, Pica R (2014). Extraintestinal manifestations in a large series of Italian inflammatory bowel disease patients. World J Gastroenterol.

[10] Greuter T, Navarini A, Vavricka SR (2017). Skin Manifestations of Inflammatory Bowel Disease. Clin Rev Allergy Immunol.

[11] Antonelli E, Bassotti G, Tramontana M (2021). Dermatological Manifestations in Inflammatory Bowel Diseases. J Clin Med.

[12] Garcovich S, De Simone C, Genovese G, Berti E, Cugno M, Marzano AV (2019). Paradoxical Skin Reactions to Biologics in Patients With Rheumatologic Disorders. Front Pharmacol.

[13] Nigam GB, Bhandare AP, Antoniou GA, Limdi JK (2021). Systematic review and meta-analysis of dermatological reactions in patients with inflammatory bowel disease treated with anti-tumour necrosis factor therapy. Eur J Gastroenterol Hepatol.

[14] Russell MD, Stovin C, Alveyn E (2023). JAK inhibitors and the risk of malignancy: a meta-analysis across disease indications. Ann Rheum Dis.

[15] Curtis JR, Yamaoka K, Chen YH (2023). Malignancy risk with tofacitinib versus TNF inhibitors in rheumatoid arthritis: results from the open-label, randomised controlled ORAL Surveillance trial. Ann Rheum Dis.

[16] Weizman AV, Sharma R, Afzal NM (2018). Stricturing and Fistulizing Crohn’s Disease Is Associated with Anti-tumor Necrosis Factor-Induced Psoriasis in Patients with Inflammatory Bowel Disease. Dig Dis Sci.

[17] Laranjeira N, Fonseca J, Meira T, Freitas J, Valido S, Leitão J (2015). Oral mucosa lesions and oral symptoms in inflammatory bowel disease patients. Arq Gastroenterol.

[18] Ampuero J, Rojas-Feria M, Castro-Fernández M, Cano C, Romero-Gómez M (2014). Predictive factors for erythema nodosum and pyoderma gangrenosum in inflammatory bowel disease. J Gastroenterol Hepatol.

[19] Alinaghi F, Tekin HG, Burisch J, Wu JJ, Thyssen JP, Egeberg A (2020). Global Prevalence and Bidirectional Association Between Psoriasis and Inflammatory Bowel Disease-A Systematic Review and Meta-analysis. J Crohns Colitis.

[20] Denadai R, Teixeira FV, Steinwurz F, Romiti R, Saad-Hossne R (2013). Induction or exacerbation of psoriatic lesions during anti-TNF-α therapy for inflammatory bowel disease: a systematic literature review based on 222 cases. J Crohns Colitis.

[21] Cleynen I, Van Moerkercke W, Billiet T (2016). Characteristics of Skin Lesions Associated With Anti-Tumor Necrosis Factor Therapy in Patients With Inflammatory Bowel Disease: A Cohort Study. Ann Intern Med.

[22] Au M, Heddle G, Young E (2023). Anti-tumour necrosis factor-induced skin rashes in inflammatory bowel disease: a systematic review and evidence-based management algorithm. Intern Med J.

